# Estimating Light Acclimation Parameters of Cucumber Leaves Using Time-Weighted Averages of Daily Photosynthetic Photon Flux Density

**DOI:** 10.3389/fpls.2021.809046

**Published:** 2022-02-08

**Authors:** Liyao Yu, Kazuhiro Fujiwara, Ryo Matsuda

**Affiliations:** Department of Biological and Environmental Engineering, Graduate School of Agricultural and Life Sciences, The University of Tokyo, Tokyo, Japan

**Keywords:** chlorophyll *a/b* ratio, photosynthetic capacity, regression model, leaf mass per area, photosynthetic photon flux density, light emitting diode

## Abstract

Leaves acclimate to day-to-day fluctuating levels of photosynthetic photon flux density (PPFD) by adjusting their morphological and physiological parameters. Accurate estimation of these parameters under day-to-day fluctuating PPFD conditions benefits crop growth modeling and light environment management in greenhouses, although it remains challenging. We quantified the relationships between day-to-day PPFD changes over 6 days and light acclimation parameters for cucumber seedling leaves, including leaf mass per area (LMA), chlorophyll (Chl) *a*/*b* ratio, maximum net photosynthetic rate (*P*_nmax_), maximum rate of ribulose-1,5-bisphosphate (RuBP) carboxylase/oxygenase (*V*_cmax_), and maximum rate of electron transport (*J*_max_). The last two parameters reflect the capacity of the photosynthetic partial reactions. We built linear regression models of these parameters based on average or time-weighted averages of daily PPFDs. For time-weighted averages of daily PPFDs, the influence of daily PPFD was given a specific weight. We employed three types of functions to calculate this weight, including linear, quadratic, and sigmoid derivative types. We then determined the trend of weights that estimated each parameter most accurately. Moreover, we introduced saturating functions to calibrate the average or time-weighted averages of daily PPFDs, considering that light acclimation parameters are usually saturated under high PPFDs. We found that time-weighted average PPFDs, in which recent PPFD levels had larger weights than earlier levels, better estimated LMA than average PPFDs. This suggests that recent PPFDs contribute more to LMA than earlier PPFDs. Except for the Chl *a*/*b* ratio, the average PPFDs estimated *P*_nmax_, *V*_cmax_, and *J*_max_ with acceptable accuracy. In contrast, time-weighted averages of daily PPFDs did not improve the estimation accuracy of these four parameters, possibly due to their low response rates and plasticity. Calibrating functions generally improved estimation of Chl *a*/*b* ratio, *V*_cmax_, and *J*_max_ because of their saturating tendencies under high PPFDs. Our findings provide a reasonable approach to quantifying the extent to which the leaves acclimate to day-to-day fluctuating PPFDs, especially the extent of LMA.

## Introduction

The photosynthetic photon flux density (PPFD) of sunlight fluctuates at various time scales, from seconds to months. Plant leaves can sense fluctuations in PPFD and adjust their foliar properties to better fit the changing PPFD levels. This adjustment that occurs in the long term (e.g., from days to months) has been defined as light acclimation and is thought to be beneficial for leaf growth under given light environments. For example, leaves acclimated to low PPFD levels tend to show a lower leaf mass per area (LMA) and thickness (Björkman and Holmgren, 1963; Clough et al., 1979), a lower chlorophyll (Chl) *a*/*b* ratio (Boardman, 1977; Rozendaal et al., 2006), and a lower maximum net photosynthetic rate (*P*_nmax_) (McCree and Troughton, 1966). These traits contribute to increasing light interception and absorption per leaf and photosynthesis, with the efficient use of nitrogen in the photosynthetic components (Field, 1983). Conversely, leaves acclimated to high PPFD levels tend to be thick and have a high LMA (Boardman, 1977; Wu et al., 2018), a high Chl *a/b* ratio (Boardman, 1977; Kitajima and Hogan, 2003), and high *P*_nmax_ (McCree and Troughton, 1966). These properties are beneficial to leaves under high PPFD levels because of a higher photosynthetic rate per leaf area and less possibility of high light damage (Szechyńska-Hebda et al., 2010; Talhouët et al., 2020). These light acclimation responses are important not only to wild species in natural habitats, but also to crops cultivated in open fields and greenhouses.

Although the morphological and physiological parameters of leaves acclimated to the aforementioned contrasting PPFDs are well known, how plants utilize previously experienced PPFD information to alter these parameters remains poorly understood. In particular, the quantitative link between PPFD changes and the extent of acclimation responses has not been fully determined. Quantifying this link would be of help not only to understand the physiological mechanism of light acclimation, but also to improve the crop growth models (e.g., Jones et al., 1991, 2003; Heuvelink, 2005; Yin and van Laar, 2005; Wu et al., 2016) to better predict growth and yield. In addition, this quantification can contribute to improving light environmental management in greenhouses. Most studies estimating the extent of light acclimation responses have been conducted in wild vegetation and open fields or greenhouses using fluctuating sunlight PPFD (Valladares et al., 2000; Dias et al., 2018; Baer et al., 2020; Deguchi and Koyama, 2020). The problem with this approach is that environmental factors other than PPFD, such as light spectrum, temperature, and wind, cannot be fully controlled. Thus, experimental reproducibility is not ensured. However, growth chamber experiments with artificial lighting can solve this problem (Vialet-Chabrand et al., 2017; Flannery et al., 2021; Wei et al., 2021). Moreover, in most cases the PPFD levels in growth chambers during the light period are set at a constant level throughout the experiment and do not fluctuate, as does those of sunlight. In our previous study (Yu et al., 2022), cucumber seedlings were grown in growth chambers, and their leaves were treated with day-to-day changing PPFDs (constant within each light period). Light was provided by white light-emitting diodes (LEDs). We evaluated the LMA, Chl *a*/*b* ratio, maximum rate of ribulose-1,5-bisphosphate carboxylase/oxygenase (*V*_cmax_), and maximum rate of electron transport (*J*_max_). We found that time-weighted averages of daily PPFD estimated the extent of light acclimation responses better than the simple average PPFD. This strongly suggests that the extent of light acclimation responses can be quantitatively estimated by appropriately incorporating the characteristics of day-to-day PPFD changes.

However, there are several aspects of our previous study that required improvement. First, leaves experienced three daily PPFD levels, each lasting for 2 days. Treatments with randomly assigned PPFD levels during the light period per day would help validate our models under more distinctly day-to-day changing PPFD levels. Second, to assign an appropriate weight to each of the daily PPFD levels, we tested four types of weight functions: linear, exponential, quadratic, and saturating. All functions showed similar accuracy in estimating LMA and Chl *a*/*b* ratio. A possible reason could be that although these functions vary in their trend shapes, they are all monotonic and reach their maximum on the day before the measurement. We hypothesized that plants have “fading memories” for PPFD levels experienced over time. This assumption might be appropriate for the LMA, which is considered highly plastic (Poorter et al., 2009), and to respond quickly to PPFD changes (Noguchi et al., 2001; Zhang et al., 2017), especially for young growing leaves. Conversely, for other parameters such as Chl *a*/*b* ratio, which usually responds at a lower rate and plasticity (De la Torre and Burkey, 1990; Trouwborst et al., 2010), it is physiologically reasonable that a clear response takes several days (Turnbull et al., 1993). In this situation, the possibility that the influence of daily PPFD peaks in several days before the measurement, rather than a day before, needs to be tested.

Therefore, the purpose of this study was to estimate leaf parameters of acclimation responses using linear regression models on time-weighted averages of daily PPFDs and to determine the weight-per-day trend, which reasonably estimated these parameters when leaves were subjected to day-to-day PPFD changes. We conducted two experiments, both with 6-day PPFD treatments. In the first experiment, daily PPFD in the light period per day was randomly assigned in the range of 100–700 μmol m^–2^ s^–1^. In the second experiment, leaves were subjected to treatments in which a PPFD level different from the basal PPFD levels was assigned to only 1 day, either the fourth, fifth, or sixth day. The reason why treatments with a different PPFD on the first, second, or third day were not included was based on the assumption that the PPFD levels in the early half of the treatment period are not likely to contribute more than those in the later half. We evaluated light acclimation response parameters, including LMA, Chl *a*/*b* ratio, *P*_nmax_, *V*_cmax_, and *J*_max_. We built linear regression models of these parameters based on average or time-weighted averages of daily PPFDs. To test whether weight functions with a peak several days before the measurement could better estimate these parameters, we calculated time-weighted averages of daily PPFDs with one of the three types of weight functions: linear, quadratic, or sigmoid derivative—the last two functions can be convex-upward with a peak. Finally, considering that leaf acclimation parameters usually saturate under high PPFDs, we tested whether referring this information by calibrating average or time-weighted averages of daily PPFDs *via* saturating functions prior to building the estimation models further improved the accuracy.

## Materials and Methods

### Plant Material and Growth Conditions

Seeds of cucumber (*Cucumis sativus* L.) cultivar “Hokushin” (Takii Co., Ltd., Kyoto, Japan) were sown in plug trays filled with commercial rockwool cubes (Grodan Delta, Grodan, Roermond, Netherlands) and were grown in temperature-controlled growth chambers (MIR-553, Sanyo Electric Co., Ltd., Osaka, Japan). Phosphor-converted white LED (GSPW1651NSE-E0Y-YPW Stanley Electric Co., Ltd., Tokyo, Japan; spectral distribution of photon flux density is shown in Supplementary Figure 1) panels were placed in the growth chambers as the light source.

The environment in the growth chambers was set as follows: PPFD was measured at the level of the upper surface of the rockwool cubes, 15 cm below the LED panel, using a light quantum sensor (LI-190R, Li-Cor Inc., Lincoln, NE, United States) set at 300 μmol m^–2^ s^–1^; daily light/dark periods were 16/8 h; air temperatures were 25°C for the light period and 20°C for the dark period. Ambient air was continuously pumped into the growth chambers at a flow rate of 6 L min^–1^ using air pumps (APN-100R, Iwaki Co., Ltd., Tokyo, Japan) to maintain a CO_2_ concentration of ∼400 μmol mol^–1^. The relative humidity was 70% or higher throughout the day. Rockwool cubes were irrigated with 2 L of tap water in plastic cultivation trays (40 × 32 × 7.5 cm) until the seeds were germinated, and then with 2 L of commercial nutrient solution (OAT prescription A, OAT Agrio Co., Ltd., Tokyo, Japan) with an electrical conductivity of 190 mS m^–1^ and pH of 6.4 every 2 days. Once the seeds were germinated, the distance between the LED panels and seedling cotyledons was kept at 15 cm by adjusting the distance between the LED panels and the cultivation tray every 2 days.

### Treatments

The first true seedling leaves were subjected to treatments at the end of the 10th day after sowing, when they were fully unfolded and had started to expand. The distance between the LED panels and the leaves was maintained at 15 cm. The environmental conditions, other than PPFD, in the treatment period were the same as those for the seedling-raising period. For both experiments, four plants were treated from the 11th to the 16th day after sowing, and three plants were collected for measurement on the 17th day after sowing per treatment. In the first experiment, plants were subjected to 12 PPFD treatments for 6 days. The PPFD level during the light period was held constant at 100, 200, 300, 400, 500, 600, or 700 μmol m^–2^ s^–1^. One of these seven PPFD levels was randomly assigned in each light period of the 6 days of treatment. The treatment numbers and details are presented in Supplementary Table 1. Due to our limited experimental capacity, these 12 treatments were not conducted simultaneously, but were divided into two cultivation experiments. Each cultivation experiment comprised of 6 of these 12 treatments and a common treatment with a PPFD of 100 μmol m^–2^ s^–1^ (treatment CL) in all the light periods. In the two cultivation experiments, CL treatment served as the standard for data relativization. For example, the relative LMA of a treatment was calculated as the ratio of the absolute value of LMA for that treatment to that of the CL treatment.

In the second experiment, the PPFD level during the light period in 5 days of the 6-day period was held constant as the basal PPFD level, and was one of the three PPFD levels of 100, 400, and 700 μmol m^–2^ s^–1^. For each treatment, one of these three PPFD levels, which differed from the basal PPFD level, was assigned on either the fourth, fifth, or sixth day of the period. The details of the PPFD setting for all the treatments are shown in Supplementary Table 2. The environmental conditions, other than PPFD, were the same as those used in the first experiment. All treatments were evenly divided into three cultivation experiments, each of which contained six treatments in addition to the two reference treatments with basal PPFD levels per light period in all the 6 days. In the two former cultivation experiments, treatment CL served as the standard for data relativization. In the last cultivation experiment, CL was not included due to our limited experimental capacity. Reference treatment CM (with a constant PPFD of 400 μmol m^–2^ s^–1^ in the light period per day) served as the primary standard of relativization; data in this cultivation experiment were relativized as the ratio of the measured values of each treatment to that of the treatment CM, multiplied by the ratio of those of treatment CM to treatment CL in the first cultivation experiment.

### Leaf Mass Per Area and Chl *a/b* Ratio Measurement

The methods used to measure the LMA and Chl *a*/*b* ratio were the same as those used by Yu et al. (2022). The LMA was calculated as the ratio of leaf dry mass to leaf area (*n* = 3). The Chl *a* and *b* content per leaf area was determined, and the molar Chl *a*/*b* ratio was calculated according to the method of Porra et al. (1989) (*n* = 3).

### Gas Exchange and CO_2_ Response Curve Measurements

We determined the leaf photosynthetic CO_2_ response curves and estimated parameters including *P*_nmax_, *V*_cmax_, and *J*_max_, according to the FvCB biochemical model of photosynthesis (Farquhar et al., 1980; Sharkey, 1985). This model demonstrates that the net photosynthetic rate is limited by a minimum of three rates: *V*_cmax_, *J*_max_, or the rate of triose-phosphate utilization (TPU). We did not consider TPU parameter because the TPU limitation requires a high intercellular CO_2_ concentration (von Caemmerer, 2000), which was unlikely to occur in our cultivation environment. The method used to measure these parameters was similar to that of Yu et al. (2022), with some minor changes. Photosynthetic CO_2_ response curves of the first true leaves were measured on the day after the treatment period (the 17th day after sowing) using a portable photosynthesis measurement system (LI-6800 FP/M, LiPCor Inc.), according to the rapid *A*-*C*_*i*_ response (RACiR) method (Stinziano et al., 2017) (*n* = 3). In the RACiR method, the CO_2_ concentration in the reference chamber ([CO_2_]_Ref_) is changed continuously to determine the leaf photosynthetic CO_2_ response curve, a faster method than the conventional steady-state curve method. In a preliminary experiment with nine leaves, the differences in estimated values of *V*_cmax_ and *J*_max_ *via* the RACiR and steady-state methods were not significantly different (paired *t*-test, *p >* 0.05, *n* = 9). The chamber conditions for the measurements were as follows: a gas flow rate of 600 μmol s^–1^, a leaf-to-air vapor pressure deficit of 1.5 kPa, a leaf temperature of 25°C, and a saturating PPFD of 1,800 μmol m^–2^ s^–1^, in which light was provided by a red and blue LED light source (3 × 3 cm, 6800- 2P, red/blue PPFD ratio of 9:1, Lie-Cor Inc.). A point-matching procedure for infrared gas analyzers (IRGAs) was conducted under [CO_2_]_Ref_ at 400 μmol mol^–1^ before performing RACiR. Before measurement, each leaf was acclimated in [CO_2_]_Ref_ at 400 μmol mol^–1^ and the above-mentioned chamber conditions for at least 10 min to stabilize the leaf net photosynthesis and transpiration. Subsequently, [CO_2_]_Ref_ was linearly increased from 200 to 1,000 μmol mol^–1^ over 8 min (at a rate of 100 μmol mol^–1^ min^–1^), during which the gas exchange parameters were logged every 2 s. To eliminate the effects of systematic residual delays during the linear change of [CO_2_]_Ref_, chamber leakage and match offsets of IRGAs, and an additional CO_2_ response curve was acquired using the empty leaf chamber under the same chamber conditions, as shown in Supplementary Figure 2. Calibrated leaf CO_2_ response curves were fitted using the FvCB model. *P*_nmax_ at an ambient CO_2_ concentration (*C*_a_) of 400 μmol mol^–1^ and at an intercellular CO_2_ concentration (*C*_i_) of 400 μmol mol^–1^, *V*_cmax_, and *J*_max_ were then estimated using the R package “plantecophys” (ver. 1.4-4, Duursma, 2015) in R software (ver. 3.6.2, R Core Team). The mean of coefficient of determination (*R*^2^) for a fitting exercise was 0.99 (121 leaves).

### Model Construction

We considered the time-weighted averages of daily PPFD (*Q*_w_), in which each daily PPFD was weighted depending on how much it contributed to the light acclimation parameters. The influence of PPFD on and before the 10th day after sowing was not considered since our previous study (Yu et al., 2022) indicated that its contributions were small enough to be negligible. *Q*_w_ was then calculated as the accumulated product of the daily PPFD and its weight, divided by the accumulated weights. Thus, the function of *Q*_w_ is defined as follows:


(1) $$Q_{w}=\frac{\sum_{t=1}^{6}\left[Q\,\left(t\right)\times W\,\left(t\right)\right]}{\sum W\,\left(t\right)}$$

where *t* is the number of days after treatment began (*t* = 1, 2, …, 6), *Q*(*t*) is the PPFD on day *t*, and *W*(*t*) is the weight of PPFD on day *t*. On the other hand, the average PPFD (*Q*_m_) was calculated assuming *W*(*t*) was constant irrespective of *t*:


(2) $$Q_{m}=\frac{\sum_{t=1}^{6}Q\,\left(t\right)}{t}$$

The function of *W*(*t*) for the linear type of *Q*_w_ (*Q*_wl_), *W*_l_(*t*), is defined as follows:


(3) $$W_{l}\left(t\right)=\mathrm{at}+b\left(a\neq 0\right)$$

The functions of *W*(*t*) for the quadratic and sigmoid derivative types of *Q*_w_ (*Q*_wq_ and *Q*_wsd_), *W*_q_(*t*), and *W*_sd_(*t*) are defined as follows:


(4) $$W_{q}\left(t\right)={\mathrm{ct}}^{2}+\mathrm{dt}+f\left(c\neq 0\right)$$

(5) $$W_{s\,d}\left(t\right)=\frac{{\mathrm{ge}}^{-g\,\left(t+h\right)}}{{\left(1+e^{-g\,\left(t+h\right)}\right)}^{2}}\left(g>0\right)$$

Two other types of *Q*_w_ were introduced from our previous study: exponential (*Q*_we_) and saturating type (*Q*_ws_). Their *W*(*t*), *W*_e_(*t*), and *W*_s_(*t*) were calculated as follows:


(6) $$W_{e}\left(t\right)=i^{\left(t+j\right)}+k\left(i>1\right)$$

(7) $$W_{s}\left(t\right)=1-\frac{1}{l^{\left(\mathrm{mt}+n\right)}}\left(l>1\right)$$

We calibrated *Q*_m_ and *Q*_w_ *via* asymptotic functions. In an asymptotic function adopted from Poorter et al. (2019), the dependent variable approaches a horizontal asymptote in a saturating manner when the independent variable increases. Therefore, the calibration factors *CF*(*Q*[*t*]) were defined as:


(8) $$\mathrm{CF}\left(Q\left[t\right]\right)=p\left(1-{\mathrm{qe}}^{-\mathrm{rQ}\,\left(t\right)}\right)\left(p,q,r>0\right)$$

The calibrated PPFD on day *t* (*CQ*[*t*]) was calculated as:


(9) CQ⁢(t)=Q⁢(t)×CF⁢(Q⁢[t])

Thus, the calibrated *Q*_w_ (*CQ*_w_) and calibrated *Q*_m_ (*CQ*_m_) were defined as:


(10) $${\mathrm{CQ}}_{w}=\frac{\sum_{t=1}^{6}\left[\mathrm{CQ}\,\left(t\right)\times W\,\left(t\right)\right]}{\sum_{t=1}^{6}W\,\left(t\right)}$$

(11) $${\mathrm{CQ}}_{m}=\frac{\sum_{t=1}^{6}\mathrm{CQ}\,\left(t\right)}{t}$$

The three types of *CQ*_w_ (*Q*_wl_, *Q*_wq_, and *Q*_wsd_) were designated *CQ*_wl_, *CQ*_wq_, and *CQ*_wsd_, respectively.

To determine the best-fit model parameters (*a*–*r* in Equations 3–8) for estimating LMA, Chl *a/b* ratio, *P*_nmax_, *V*_cmax_, or *J*_max_ based on *Q*_m_, *Q*_w_, *CQ*_m_, or *CQ*_w_, we set the initial values to parameters in each weight function and calculated the *Q*_w_ or *CQ*_w_. Then we built the linear regression model of each estimated light acclimation parameter using the least-squares method and calculated the root mean squared error (RMSE). At last, we employed the optimization routine to find the parameters that gave the minimum RMSE.

### Statistics

All data were presented as means ± standard errors from three biological replicates. To unbiasedly compare regression models with different quantities of variables, we calculated the adjusted coefficient of determination of regression (*$\bar{R}$*^2^) per model using the degrees of freedom per model. The statistical significance of linear regression models was examined using the *F*-test (*p* < 0.05) in R software.

## Results

### Leaf Acclimation Responses to Constant Photosynthetic Photon Flux Density Levels

**FIGURE 1:**
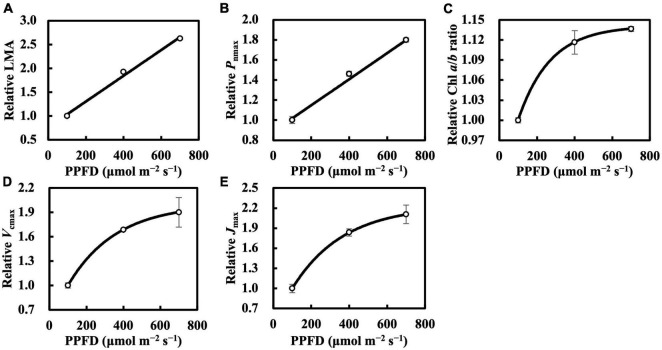
Relative leaf mass per area (LMA) **(A)**, maximum net photosynthetic rate (*P*_nmax_) at *C*_a_ of 400 μmol mol^–1^ **(B)**, chlorophyll (Chl) *a*/*b* ratio **(C)**, maximum rate of ribulose-1,5-bisphosphate carboxylase/oxygenase (*V*_cmax_) **(D)**, and maximum rate of electron transport (*J*_max_) **(E)** of the first true leaves of cucumber seedlings grown under constant daily photosynthetic photon flux density (PPFD) levels for 6 days. All values were means ± standard errors (*n* = 3) and relativized to that for 100 μmol m^–2^ s^–1^. Measured values (open circles) and estimated values (solid line) fitted with linear **(A,B)** or asymptotic **(C–E)** functions are shown. See section “Statistics” for details of asymptotic functions.

Leaf mass per area and *P*_nmax_ responded almost linearly toward constant daily PPFD levels (Figures 1A,B), whereas Chl *a*/*b* ratio, *V*_cmax_, and *J*_max_ clearly saturated at high constant daily PPFD levels (Figures 1C–E). Therefore, we calibrated *Q*_m_ and *Q*_w_ using asymptotic functions for the estimation models of the Chl *a*/*b* ratio, *V*_cmax_, and *J*_max_.

### Leaf Mass Per Area in Response to Day-to-Day Photosynthetic Photon Flux Density Changes

**FIGURE 2:**
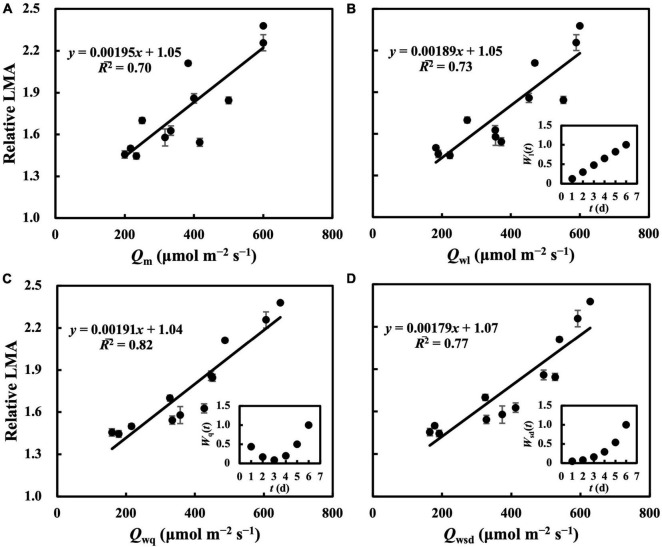
Linear regression of relative LMA on average PPFD (*Q*_m_) **(A)**, time-weighted average PPFD in linear type (*Q*_wl_) **(B)**, in quadratic type (*Q*_wq_) **(C)**, in sigmoid derivative type (*Q*_wsd_) **(D)**. First true leaves of cucumber seedlings were acclimated to randomly assigned PPFD treatments in the first experiment. All values were means ± standard errors (*n* = 3) and relativized to that for 100 μmol m^–2^ s^–1^. Regression lines, regression functions, and *$\bar{R}$*^2^ are shown. Corresponding weights of *Q*_wl_ [*W*_l_(*t*)], *Q*_wq_ [*W*_q_(*t*)], *Q*_wsd_ [*W*_sd_(*t*)] in *t* day which achieved the minimum RMSEs of estimation of LMA are shown. See section “Statistics” for details of calculation.

**FIGURE 3:**
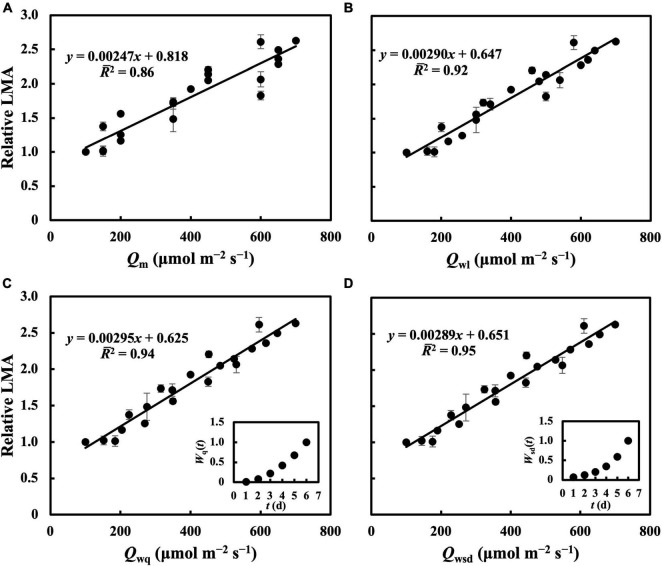
Linear regression of relative LMA on *Q*_m_ **(A)**, *Q*_wl_ **(B)**, *Q*_wq_ **(C)**, *Q*_wsd_ **(D)**. First true leaves of cucumber seedlings were acclimated to treatments in which 1 day had a PPFD level from basal PPFD levels in other 5 days in the second experiment. All values were means ± standard errors (*n* = 3) and relativized to that for 100 μmol m^–2^ s^–1^. Regression lines, regression functions, and *$\bar{R}$*^2^ are shown. Corresponding weights of *Q*_wq_ [*W*_q_(*t*)], *Q*_wsd_ [*W*_sd_(*t*)] in *t* day which achieved the minimum RMSEs of estimation of LMA are shown.

In the first experiment, the linear regression of LMA on *Q*_m_ (Figure 2A) gave a *$\bar{R}$*^2^ of 0.70 (Table 1), indicating that, to some extent, *Q*_m_ can estimate the LMA. The linear regression on *Q*_w_ (either *Q*_wl_, *Q*_wq_, or *Q*_wsd_, in Figures 2B–D) showed higher *$\bar{R}$*^2^s (0.73, 0.82, and 0.77, respectively) than that on *Q*_m_. *$\bar{R}$*^2^s (Table 1) and RMSEs (Supplementary Table 3) from the linear regression of LMA on *Q*_we_ and *Q*_ws_ were comparable to those of other *Q*_w_ types. However, because their weight trends could be similarly fitted with quadratic or sigmoid derivative functions and not vice versa, they were omitted. The weight of daily PPFD for calculating *Q*_wl_, *Q*_wq_, and *Q*_wsd_ is shown in Figures 2B–D. While the curves of weights in *Q*_wl_ and *Q*_wsd_ were monotonic, weights in *Q*_wq_ showed a down-convex curve and had their minimum on the third day. In the second experiment, models with *Q*_wl_, *Q*_wq_, or *Q*_wsd_ (Figure 3) estimated LMA with *$\bar{R}$*^2^s higher than that with *Q*_m_ (Table 1). These results show a trend approximating that of the first experiment. Time-weighted averages of daily PPFD generally estimated the LMA with higher accuracy than the average PPFD.

**TABLE 1:** Coefficient of determination (*$\bar{R}$*^2^) from regression models of light acclimation parameters of cucumber leaves on the average of daily photosynthetic photon flux density (PPFD) (*Q*_m_), time-weighted averages of daily PPFD (*Q*_w_^z^), calibrated *Q*_m_ (*CQ*_m_ *^y^*), and calibrated *Q*_w_ (*CQ*_w_ ^y^).

Parameters^x^	*$\bar{\text{R}}$*^2^ in the first experiment								*$\bar{R}$*^2^ in the second experiment							
	*Q*_m_	*Q*_wl_	*Q*_wq_	*Q*_wsd_	*CQ*_m_	*CQ*_wl_	*CQ*_wq_	*CQ*_wsd_	*Q*_m_	*Q*_wl_	*Q*_wq_	*Q*_wsd_	*CQ*_m_	*CQ*_wl_	*CQ*_wq_	*CQ*_wsd_
LMA	0.70	0.73	0.82	0.77	–^w^	–	–	–	0.86	0.92	0.94	0.95	–	–	–	–
Chl *a*/*b* ratio	0.17	0.08	0.31	–0.03	0.11	0.06	0.05	0.04	0.74	0.73	0.71	0.71	0.84	0.83	0.82	0.82
*P*_nmax_	0.65	0.62	0.63	0.62	–	–	–	–	0.81	0.80	0.79	0.80	–	–	–	–
*V*_cmax_	0.74	0.71	0.69	0.69	0.77	0.74	0.71	0.71	0.76	0.74	0.73	0.73	0.87	0.87	0.86	0.86
*J*_max_	0.77	0.74	0.72	0.72	0.78	0.76	0.73	0.73	0.67	0.65	0.66	0.66	0.81	0.82	0.81	0.81

*^z^Q_w_ of linear (Q_wl_), quadratic (Q_wq_), sigmoid derivative (Q_wsd_), exponential (Q_we_), or saturating (Q_ws_) type were calculated with their corresponding weight functions. See section “Statistics” for the details.*

*^y^Q_m_ and Q_w_ were calibrated using an asymptotic function. See section “Statistics” for the details.*

*^x^LMA, leaf mass per area; Chl, chlorophyll; P_nmax_, maximum net photosynthetic rate; V_cmax_, maximum rate of ribulose-1,5-bisphosphate carboxylase/oxygenase; J_max_, maximum rate of electron transport.*

*^w^Q_m_ and Q_w_ for estimating the LMA and P_nmax_ were not calibrated.*

### Chl *a/b* Ratio in Response to Day-to-Day Photosynthetic Photon Flux Density Changes

**FIGURE 4:**
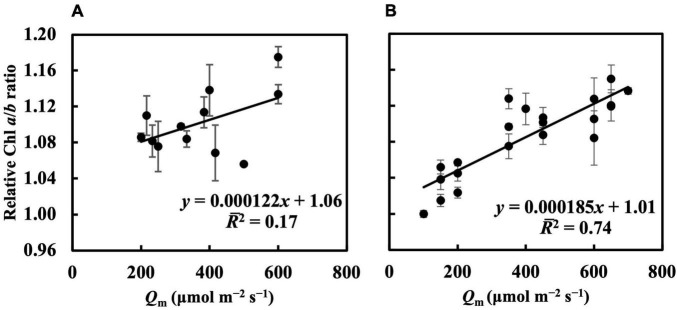
Linear regression of relative Chl *a*/*b* ratio on *Q*_m_ in the first **(A)** and second **(B)** experiment (see section “Treatments” for the details of treatment setting in each experiment). All values were means ± standard errors (*n* = 3) and relativized to that for 100 μmol m^–2^ s^–1^. Regression lines, regression functions, and *$\bar{R}$*^2^ are shown.

In the first experiment, the linear regression of the Chl *a*/*b* ratio on *Q*_m_, *Q*_wl_, and *Q*_wsd_ was not statistically significant (*p* > 0.05) (Figure 4A and Supplementary Figures 3A,B), indicating that they could not effectively estimate the Chl *a*/*b* ratio. Although linear regression of the Chl *a*/*b* ratio on *Q*_wq_ was statistically significant (*p* < 0.05, Supplementary Figure 3C), the *$\bar{R}$*^2^ (0.31) was low, implying that *Q*_wq_ could not be considered appropriate for estimating the Chl *a*/*b* ratio. Linear regression of the Chl *a*/*b* ratio on *CQ*_m_, *CQ*_wl_, *CQ*_wsd_, and *CQ*_wq_ was statistically insignificant. Therefore, the weight functions that we currently verified did not accurately estimate the Chl *a*/*b* ratio. In the second experiment, the linear regression of Chl *a*/*b* ratio on *Q*_m_ and *Q*_w_ (Figure 4B and Supplementary Figures 3D–F) had *$\bar{R}$*^2^s of 0.74, 0.73, 0.71, and 0.71, respectively (Table 1). The models of the Chl *a*/*b* ratio on *CQ*_m_, *CQ*_wl_, *CQ*_wq_, and *CQ*_wsd_ showed *$\bar{R}$*^2^s of 0.84, 0.83, 0.82, and 0.82, respectively (Table 1). *CQ*_m_ estimated the Chl *a*/*b* ratio with high accuracy, and none of the *CQ*_w_ further improved this estimation.

### *P*_nmax_, *V*_cmax_, and *J*_max_ in Response to Day-to-Day Photosynthetic Photon Flux Density changes

**FIGURE 5:**
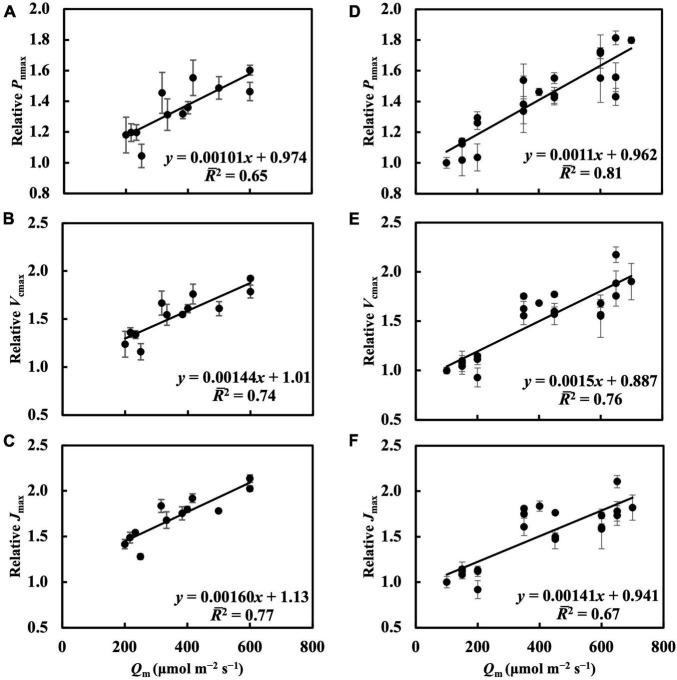
Linear regression of relative *P*_nmax_, *V*_cmax_, and *J*_max_ on *Q*_m_ in the first experiment **(A–C)** and in the second experiment **(D–F)**. See section “Treatments” for the details of treatment setting in each experiment. All values were means ± standard errors (*n* = 3) and relativized to that for 100 μmol m^–2^ s^–1^. Regression lines, regression functions, and *$\bar{R}$*^2^ are shown.

In the first experiment, the linear regression of *P*_nmax_, *V*_cmax_, and *J*_max_ on *Q*_m_ (Figures 5A–C) had *$\bar{R}$*^2^s of 0.65, 0.74, and 0.77, respectively (Table 1). Estimating *P*_nmax_ using *Q*_wl_, *Q*_wq_, or *Q*_wsd_ (Supplementary Figures 4A–C) gave similar *$\bar{R}$*^2^s (Table 1). The estimation of *V*_cmax_ (Supplementary Figure 5) and *J*_max_ (Supplementary Figure 6) showed results similar to those of *P*_nmax_, indicating that *Q*_m_ estimated these photosynthetic parameters with accuracy similar to that of *Q*_w_. *CQ*_m_, *CQ*_wl_, *CQ*_wq_, and *CQ*_wsd_ estimated *V*_cmax_ and *J*_max_ with higher *$\bar{R}$*^2^s than their uncalibrated counterparts (Table 1). In the second experiment, *Q*_m_ estimated *P*_nmax_, *V*_cmax_, and *J*_max_ (Figures 5D–F) with *$\bar{R}$*^2^s of 0.81, 0.76, and 0.67, respectively (Table 1). Again, *Q*_wl_, *Q*_wq_, or *Q*_wsd_ estimated *P*_nmax_ (Supplementary Figure 4), *V*_cmax_ (Supplementary Figure 7), and *J*_max_ (Supplementary Figure 8) with similar accuracy (Table 1). *CQ*_m_, *CQ*_wl_, *CQ*_wq_, and *CQ*_wsd_ estimated *V*_cmax_ and *J*_max_ with higher *$\bar{R}$*^2^s than their uncalibrated counterparts (Table 1). These results were consistent with those of the first experiment.

## Discussion

### Average or Time-Weighted Averages of Daily Photosynthetic Photon Flux Density: Which Estimates Leaf Mass Per Area Better?

**FIGURE 6:**
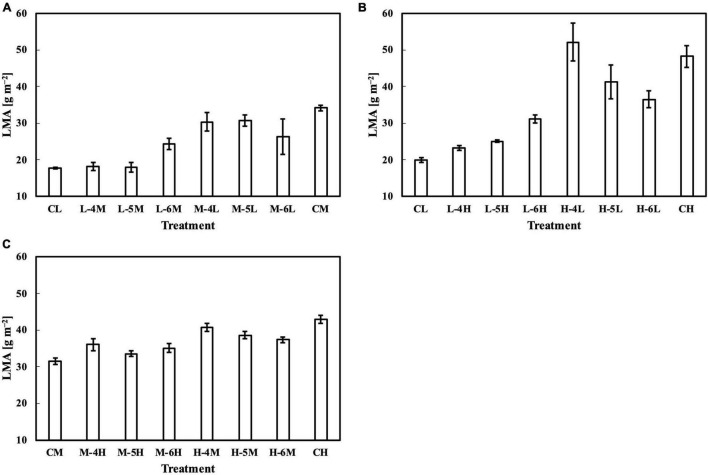
LMA of leaves subjected to a PPFD for 1 day different from the basal PPFD in the other days. CL and CM: treatments with a constant PPFD of 100 and 400 μmol m^–2^ s^–1^, respectively. The experiment was divided into three groups **(A–C)** and conducted separately due to limited experimental capacity. Values were means ± standard errors (*n* = 3). L–4M: treatment with a PPFD of 400 μmol m^–2^ s^–1^ in the fourth day of treatment period and a basal PPFD of 100 μmol m^–2^ s^–1^ in the other 5 days. See Supplementary Table 2 for details of treatments.

For leaves acclimated to constant daily PPFD, LMA responded almost linearly to daily PPFD levels (Figure 1A). This is evidence of a high plasticity and response rate, consistent with studies by Strauss-Debenedetti and Bazzaz (1991), Chazdon and Kaufmann (1993), and Poorter et al. (2019). In our first experiment, *Q*_w_, including *Q*_wl_, *Q*_wq_, and *Q*_wsd_, estimated LMA with a higher accuracy than *Q*_m_ (Figure 2), with similar findings obtained in our second experiment. All *Q*_w_ were calculated with larger weights on recent PPFD levels and smaller weights on earlier levels, except *Q*_wq_, in the first experiment, in which the minimum weight occurred on the third day of the 6-day period. However, since the *$\bar{R}$*^2^s of models on all types of *Q*_w_ were comparable, this exception should be ignored. These results are generally consistent with our previous study. Results of LMA in the second experiment (Figure 6) allowed us to test the effect of changing the daily PPFD for 1 day in the 6-day period. Accept treatment M–4H in Figure 6C, all treatments showed a common pattern that the LMA was altered the most compared to the control when the changes of daily PPFD levels occurred on the 6th day and vice versa. These results demonstrated that recent PPFD levels contributed more to LMA than the earlier levels.

Poorter et al. (2009, 2019) reviewed the literature and found that the LMA was determined by the daily light integral (DLI) or the average PPFD in a period. These reviews were based on Chabot et al. (1979) and Niinemets et al. (2004). The study by Chabot et al. (1979) on young leaves of *Fragaria virginiana* revealed that LMA was determined by the average PPFD over 9 days rather than the peak PPFD per day. However, no day-to-day changes were included in the PPFD regime in the study. On the other hand, Niinemets et al. (2004) found that LMA was highly correlated with the average PPFD for mature leaves of two shade plants when grown under sunlight. Although the LMA could generally be estimated by the simple average PPFD with good accuracy, our results provided the first clear evidence that the time-weighted averages of daily PPFD further improved the estimation of LMA for leaves under day-to-day changing PPFDs.

### *Q*_w_ Is Inferior in Estimating the Chl *a/b* Ratio and Photosynthetic Parameters Than *Q*_m_

The results of the Chl *a*/*b* ratio, *P*_nmax_, *V*_cmax_, and *J*_max_ showed that *Q*_w_ with the weight functions we tested was inferior in estimating these parameters when compared with *L*_m_. Although *CQ*_m_ could not effectively estimate Chl *a*/*b* ratio in the first experiment, it did estimate the Chl *a*/*b* ratio in the second experiment and *P*_nmax_, *V*_cmax_, and *J*_max_ in both experiments with acceptable *$\bar{R}$*^2^s. These results suggest that daily PPFD on any day during treatment has an equal influence on these parameters. The generally lower accuracy of estimating these parameters than estimating LMA could be due to their lower response rates and plasticity to day-to-day PPFD changes, especially when daily PPFD changes distinctly. This is supported by Grassi and Bagnaresi (2001) and Poorter et al. (2019), whose coefficients of determination (*R*^2^) for estimating the Chl *a*/*b* ratio using average PPFD (both *R*^2^ = 0.43) were much lower than those for LMA (*R*^2^ = 0.74 and 0.68, respectively). Contrary to our expectations, models with weights that reach their maximum several days before the measurement did not improve the estimation of the Chl *a*/*b* ratio, *P*_nmax_, *V*_cmax_, and *J*_max_, indicating that leaves likely failed to effectively respond to the interval (1 day) and/or magnitude (up to 600 μmol m^–2^ s^–1^) of PPFD changes, especially in the first experiment. Athanasiou et al. (2010) found that a 1-or-2-days elevation of daily PPFD levels from 100 to 400 μmol m^–2^ s^–1^ did not trigger a significant increase in *P*_nmax_ of Arabidopsis. This could explain why our previous study, in which the interval of PPFD changes was 2 days, showed a clearly stronger influence of the recent PPFD on the Chl *a*/*b* ratio, *V*_cmax_, and *J*_max_ than the earlier PPFD. Retkute et al. (2015) predicted that the characteristics (duration, magnitude, etc.) of fluctuating light strongly influenced leaf acclimation responses. Our findings strongly suggest that the extent of acclimation responses is affected by the characteristics of day-to-day PPFD changes.

**FIGURE 7:**
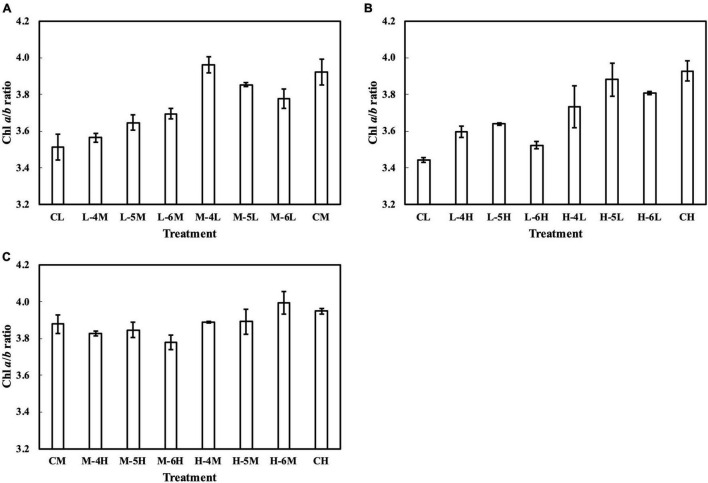
Chl *a*/*b* ratio of leaves subjected to a PPFD for 1 day different from the basal PPFD in the other days. CL and CM: treatments with a constant PPFD of 100 and 400 μmol m^–2^ s^–1^, respectively. The experiment was divided into three groups **(A–C)** and conducted separately due to limited experimental capacity. Values were means ± standard errors (*n* = 3). L–4M: treatment with a PPFD of 400 μmol m^–2^ s^–1^ in the fourth day of treatment period and a basal PPFD of 100 μmol m^–2^ s^–1^ in the other 5 days. See Supplementary Table 2 for details of treatments.

**FIGURE 8:**
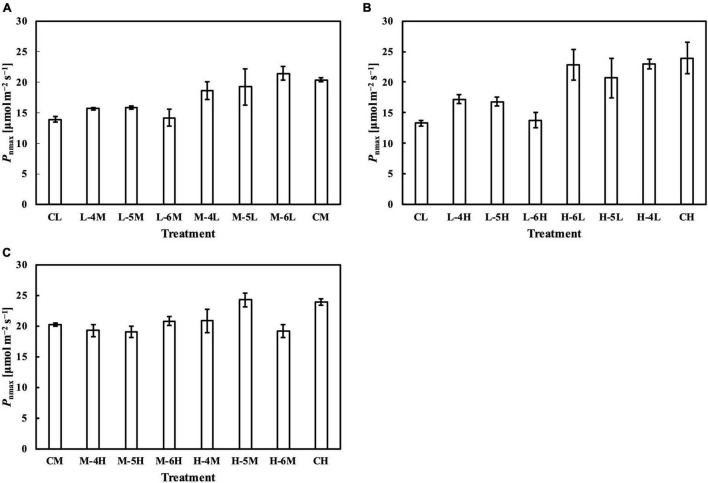
*P*_nmax_ of leaves subjected to a PPFD for 1 day different from the basal PPFD in the other days. *P*_nmax_ was measured at ambient CO_2_ concentration (*C*_a_) of 400 μmol mol^–1^ and saturating PPFD of 1,800 μmol^–1^ m^–2^ s^–1^. CL and CM: treatments with a constant PPFD of 100 and 400 μmol m^–2^ s^–1^, respectively. The experiment was divided into three groups **(A–C)** and conducted separately due to limited experimental capacity. Values were means ± standard errors (*n* = 3). L–4M: treatment with a PPFD of 400 μmol m^–2^ s^–1^ in the fourth day of treatment period and a basal PPFD of 100 μmol m^–2^ s^–1^ in the other 5 days. See Supplementary Table 2 for details of treatments.

**FIGURE 9:**
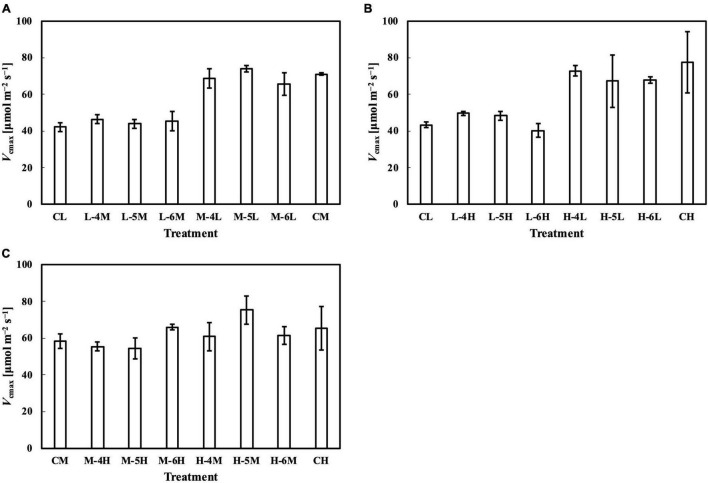
*V*_cmax_ of leaves subjected to a PPFD for 1 day different from the basal PPFD in the other days. CL and CM: treatments with a constant PPFD of 100 and 400 μmol m^–2^ s^–1^, respectively. The experiment was divided into three groups **(A–C)** and conducted separately due to limited experimental capacity. Values were means ± standard errors (*n* = 3). L–4M: treatment with a PPFD of 400 μmol m^–2^ s^–1^ in the fourth day of treatment period and a basal PPFD of 100 μmol m^–2^ s^–1^ in the other 5 days. See Supplementary Table 2 for details of treatments.

**FIGURE 10:**
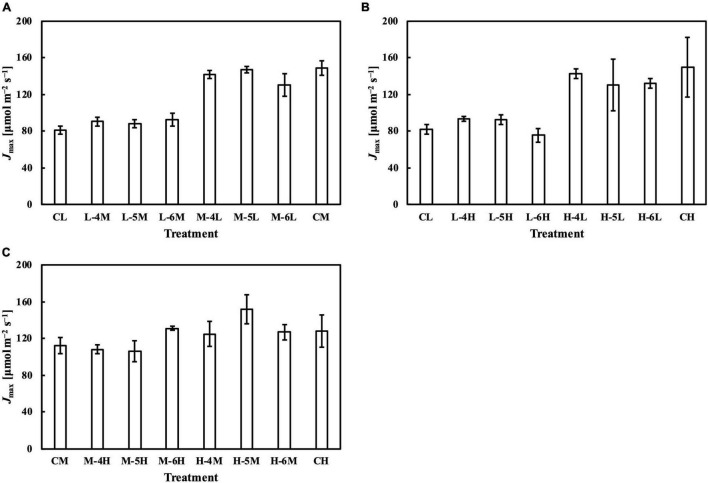
*J*_max_ of leaves subjected to a PPFD for 1 day different from the basal PPFD in the other days. CL and CM: treatments with a constant PPFD of 100 and 400 μmol m^–2^ s^–1^, respectively. The experiment was divided into three groups **(A–C)** and conducted separately due to limited experimental capacity. Values were means ± standard errors (*n* = 3). L–4M: treatment with a PPFD of 400 μmol m^–2^ s^–1^ in the fourth day of treatment period and a basal PPFD of 100 μmol m^–2^ s^–1^ in the other 5 days. See Supplementary Table 2 for details of treatments.

Meanwhile, the results of the second experiment implied that the responses of the Chl *a*/*b* ratio (Figure 7) and photosynthetic parameters (Figures 8–10) might be non-reciprocal when leaves were transferred from high to low PPFDs or vice versa. For instance, treatment M–6L had a lower *J*_max_ than treatments M–4L and M–5L (Figure 10A), suggesting that *J*_max_ was influenced by PPFD 1 day before measurement. However, no obvious difference was observed between the *J*_max_ of treatments L–4M, L–5M, and L–6M (Figure 10A), indicating a difference in light acclimation response rates in opposite directions. Although no common pattern could be observed from the comparison among all the treatments, incorporating this non-reciprocal response to day-to-day PPFD changes could improve the estimation, which should be examined in future research.

**FIGURE 11:**
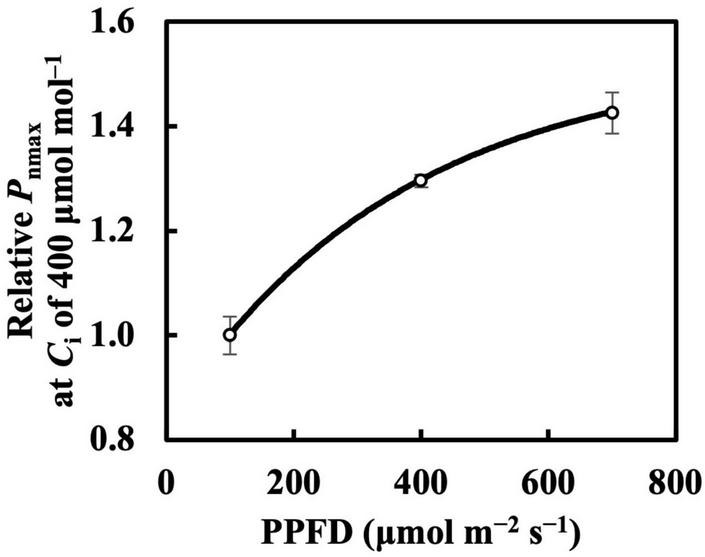
Relative *P*_nmax_ at intercellular CO_2_ concentration (*C*_i_) of 400 μmol mol^–1^ in first true leaves of cucumber grown under constant daily PPFD levels for 6 days. All values were means ± standard errors (*n* = 3) and relativized to that for 100 μmol m^–2^ s^–1^. Measured values (open circles) and estimated values (solid line) fitted with asymptotic functions are shown.

The photosynthetic capacity parameters varied in their responses to constant PPFD levels, where *P*_nmax_ at a *C*_a_ of 400 μmol mol^–1^ almost responded linearly toward PPFD levels (Figure 1B), and *V*_cmax_ and *J*_max_ were clearly saturated when PPFD levels increased (Figures 1D,E). These results suggest the existence of limiting factors for photosynthesis other than *V*_cmax_ and *J*_max_, supported, on the other hand, by the comparison in which *V*_cmax_ and *J*_max_ estimation shows higher accuracy than that of *P*_nmax_. On the other hand, when we evaluated *P*_nmax_ at a *C*_i_ of 400 μmol mol^–1^, it exhibited a clearer saturating response to PPFD levels (Figure 11). This strongly implies that leaf CO_2_ diffusion characteristics might be involved in light acclimation responses, such as stomatal properties (Mendes et al., 2001; Campany et al., 2016; Baer et al., 2020; Gjindali et al., 2021). Furthermore, a study by Björkman and Holmgren (1963) showed that leaves acclimated from high to low light for a week did not significantly differ in stomatal intensity or size compared with leaves acclimated in reverse. Thus, the limitation of stomatal properties for leaves acclimated to changing PPFD levels is indicated as reversible, rather than structurally irreversible.

### Outlook and Future Work

Daily PPFD levels might contribute equally to *P*_nmax_, *V*_cmax_, and *J*_max_, which is inconsistent with our assumption that recent PPFD levels have a larger influence on these parameters than do earlier levels. Since treatments in which 1 day in the earlier half of the 6-day period had a different PPFD from the basal level, in the other days they were not tested yet, and this meant that experiment with these treatments is needed to complete our study. Conversely, research by Luo and Keenan (2020) on the estimation of *P*_nmax_ showed that the variation in *P*_nmax_ under sunlight was better explained by the average PPFDs over the past 7 days than those over the past 10 or 15 days. Thus, the appropriate time for calculating *Q*_m_ requires further investigation.

## Conclusion

The time-weighted averages of daily PPFD estimated the LMA more accurately than the simple average PPFD, indicating that recent PPFD levels contribute more to LMA than earlier levels. Conversely, daily PPFD did not show noticeable differences in its influences on the Chl *a*/*b* ratio, *P*_nmax_, *V*_cmax_, and *J*_max_, implying that the average daily PPFD could generally estimate these parameters. The characteristics of day-to-day changes in daily PPFD were found to affect the responses of acclimation parameters. The model which appropriately incorporates the characteristics of day-to-day PPFD changes should outperform the widely used model based on an average daily PPFD in terms of estimating the LMA. Thus, our study demonstrates a reasonable approach to quantifying the extent to which leaf properties acclimate to day-to-day PPFD changes.

## Data Availability Statement

The original contributions presented in the study are included in the article/Supplementary Material, further inquiries can be directed to the corresponding author/s.

## Author Contributions

LY designed the study, conducted the experiments, analyzed the data, and wrote the manuscript. KF and RM designed the study and critically revised the manuscript. All authors contributed to the article and approved the submitted version.

## Conflict of Interest

The authors declare that the research was conducted in the absence of any commercial or financial relationships that could be construed as a potential conflict of interest.

## Publisher’s Note

All claims expressed in this article are solely those of the authors and do not necessarily represent those of their affiliated organizations, or those of the publisher, the editors and the reviewers. Any product that may be evaluated in this article, or claim that may be made by its manufacturer, is not guaranteed or endorsed by the publisher.

## Abbreviations

*C*_a_: ambient CO_2_ concentration
Chl: chlorophyll
*C*_i_: intercellular CO_2_ concentration
*CQ*_m_: calibrated average PPFD
*CQ*_wl_: calibrated time-weighted average PPFD with linear weight function
*CQ*_wq_: calibrated time-weighted average PPFD with quadratic weight function
*CQ*_wsd_: calibrated time-weighted average PPFD with derivative of sigmoid weight function
*I*_F_: forward current
IRGAs: infrared gas analyzers
*J*_max_: maximum rate of electron transport
LED: light-emitting diode
LMA: leaf mass per area
*P*_n_: net photosynthetic rate
*P*_nmax_: maximum *P*_n_ at *C*_a_ of 400 μ mol mol^–1^ and saturating PPFD of 1,800 μ mol m^–2^ s^–1^
PPFD: photosynthetic photon flux density
*Q*_m_: average PPFD
*Q*_w_: time-weighted average PPFD
*Q*_wl_: time-weighted average PPFD with linear weight function
*Q*_wq_: time-weighted average PPFD with quadratic weight function
*Q*_wsd_: time-weighted average PPFD with derivative of sigmoid weight function
*R* ^2^: coefficient of determination
* $\bar{R}$* ^2^: adjusted coefficient of determination
RMSE: root mean squared error
TPU: rate of triose-phosphate utilization
*V*_cmax_: maximum rate of carboxylation capacity of ribulose-1,5-bisphosphate carboxylase/oxygenase.
