# Supplementary material for: Estimating Light Acclimation Parameters of Cucumber Leaves Using Time-Weighted Averages of Daily Photosynthetic Photon Flux Density
Source: Front Plant Sci. 2022 Feb 8;12:809046. doi: 10.3389/fpls.2021.809046 (PMC8860900; doi:10.3389/fpls.2021.809046)
Supplement: Supplementary file 1 [file Data_Sheet_1.docx]

Supplementary Material

# Supplementary Figures and Tables

## Supplementary Figures

**Supplementary Figure 1.** Relative spectral photon flux density of light provided by white phosphor-converted LEDs at a forward current (*I*_F_) of 350 mA and an air temperature of 25°C.

**Supplementary Figure 2.** Typical response of uncalibrated net photosynthetic rate (*P*_n_) of an empty leaf chamber to the CO_2_ concentration in the reference chamber ([CO_2_]_Ref_) of LI-6800. Data between
[CO_2_]_Ref_ of 350~900 μmol mol^–1^ were used for calibrating the data deriving from leaf photosynthetic-CO_2_ response curves using the RACiR method. See 2.4 for details of the RACiR method.

**Supplementary Figure 3.** Linear regression of relative Chl *a*/*b* ratio on *Q*_wl_ (A), *Q*_wsd_ (B), and *Q*_wq_ (C) which achieved the minimum RMSEs of estimation of Chl *a*/*b* ratio for first true leaves of cucumber seedlings in the first experiment, and on *Q*_wl_ (D), *Q*_wsd_ (E), and *Q*_wq_ (F) in the second experiment. All values of Chl *a*/*b* ratio were means ± standard errors (n=3) and relativized to that for 100 μmol m^−2^ s^−1^. Regression lines, regression functions, and *R̅*^2^ are shown.

**Supplementary Figure 4.** Linear regression of relative *P*_nmax_ on *Q*_wl_ (A), *Q*_wq_ (B), *Q*_wsd_ (C) in the first experiment, and on *Q*_wl_ (D), *Q*_wq_ (E), *Q*_wsd_ (F) in the second experiment, which achieved the minimum RMSEs of estimation of *P*_nmax_ for first true leaves of cucumber seedlings. All values of *P*_nmax_ were means ± standard errors (n=3) and relativized to that for 100 μmol m^−2^ s^−1^. Regression lines, regression functions, and *R̅*^2^ are shown.

**Supplementary Figure 5.** Linear regression of relative *V*_cmax_ on *Q*_wl_ (A), *Q*_wq_ (B), *Q*_wsd_ (C), *CQ*_m_ (D), *CQ*_wl_ (E), *CQ*_wq_ (F), and *CQ*_wsd_ (G), which achieved the minimum RMSEs of estimation of *V*_cmax_ for first true leaves of cucumber seedlings in the first experiment. All values of *V*_cmax_ were means ± standard errors (n=3) and relativized to that for 100 μmol m^−2^ s^−1^. Regression lines, regression functions, and *R̅*^2^ are shown.

**Supplementary Figure 6.** Linear regression of relative *J*_max_ on *Q*_wl_ (A), *Q*_wq_ (B), *Q*_wsd_ (C), *CQ*_m_ (D), *CQ*_wl_ (E), *CQ*_wq_ (F), and *CQ*_wsd_ (G), which achieved the minimum RMSEs of estimation of *V*_cmax_ for first true leaves of cucumber seedlings in the first experiment. All values of *V*_cmax_ were means ± standard errors (n=3) and relativized to that for 100 μmol m^−2^ s^−1^. Regression lines, regression functions, and *R̅*^2^ are shown.

**Supplementary Figure 7.** Linear regression of relative *V*_cmax_ on *Q*_wl_ (A), *Q*_wq_ (B), *Q*_wsd_ (C), *CQ*_m_ (D), *CQ*_wl_ (E), *CQ*_wq_ (F), and *CQ*_wsd_ (G), which achieved the minimum RMSEs of estimation of *J*_max_ for first true leaves of cucumber seedlings in the second experiment. All values of *J*_max_ were means ± standard errors (n=3) and relativized to that for 100 μmol m^−2^ s^−1^. Regression lines, regression functions, and *R̅*^2^ are shown.

**Supplementary Figure 8.** Linear regression of relative *J*_max_ on *Q*_wl_ (A), *Q*_wq_ (B), *Q*_wsd_ (C), *CQ*_m_ (D), *CQ*_wl_ (E), *CQ*_wq_ (F), and *CQ*_wsd_ (G), which achieved the minimum RMSEs of estimation of *J*_max_ for first true leaves of cucumber seedlings in the second experiment. All values of *J*_max_ were means ± standard errors (n=3) and relativized to that for 100 μmol m^−2^ s^−1^. Regression lines, regression functions, and *R̅*^2^ are shown.

## Supplementary Tables

| **SUPP. TABLE 1 \|** Setting of the daily PPFD levels for the 6-days treatments in the first experiment | | | | | | | |
| --- | --- | --- | --- | --- | --- | --- | --- |
| Cultivation experiment | Treatment code | Daily PPFD^z^ [μmol m^−2^ s^−1^] | | | | | |
|  |  | Day1^y^ | Day2 | Day3 | Day4 | Day5 | Day6 |
| 1 | 1 | 400 | 200 | 200 | 200 | 100 | 200 |
|  | 2 | 100 | 400 | 100 | 300 | 200 | 100 |
|  | 3 | 100 | 400 | 300 | 200 | 300 | 100 |
|  | 4 | 300 | 200 | 100 | 300 | 100 | 500 |
|  | 5 | 300 | 100 | 400 | 100 | 700 | 300 |
|  | 6 | 500 | 100 | 300 | 300 | 200 | 600 |
| 2 | 7 | 300 | 100 | 600 | 600 | 100 | 700 |
|  | 8 | 400 | 500 | 700 | 400 | 200 | 300 |
|  | 9 | 200 | 300 | 100 | 600 | 400 | 700 |
|  | 10 | 100 | 600 | 500 | 700 | 700 | 400 |
|  | 11 | 700 | 600 | 500 | 500 | 600 | 700 |
|  | 12 | 700 | 500 | 700 | 500 | 600 | 600 |
| ^z^ See Supp Fig. 1 for the relative spectral photon flux density of white LED light.  ^y^ The 1st day in the treatment period and the 11th day after sowing. | | | | | | | |

| **SUPP. TABLE 2 \|** Setting of the daily PPFD levels for the 6-days treatments in the second experiment | | | | | | | |
| --- | --- | --- | --- | --- | --- | --- | --- |
| Cultivation experiment | Treatment code ^z^ | Daily PPFD [μmol m^−2^ s^−1^] | | | | | |
|  |  | Day1 | Day2 | Day3 | Day4 | Day5 | Day6 |
| 1 | L–6H ^y^ | 100 | 100 | 100 | 100 | 100 | 700 |
|  | L–5H | 100 | 100 | 100 | 100 | 700 | 100 |
|  | L–4H | 100 | 100 | 100 | 700 | 100 | 100 |
|  | H–6L | 700 | 700 | 700 | 700 | 700 | 100 |
|  | H–5L | 700 | 700 | 700 | 700 | 100 | 700 |
|  | H–4L | 700 | 700 | 700 | 100 | 700 | 700 |
| 2 | M–6L | 400 | 400 | 400 | 400 | 400 | 100 |
|  | M–5L | 400 | 400 | 400 | 400 | 100 | 400 |
|  | M–4L | 400 | 400 | 400 | 100 | 400 | 400 |
|  | L–6M | 100 | 100 | 100 | 100 | 100 | 400 |
|  | L–5M | 100 | 100 | 100 | 100 | 400 | 100 |
|  | L–4M | 100 | 100 | 100 | 400 | 100 | 100 |
| 3 | M–6H | 400 | 400 | 400 | 400 | 400 | 700 |
|  | M–5H | 400 | 400 | 400 | 400 | 700 | 400 |
|  | M–4H | 400 | 400 | 400 | 700 | 400 | 400 |
|  | H–6M | 700 | 700 | 700 | 700 | 700 | 400 |
|  | H–5M | 700 | 700 | 700 | 700 | 400 | 700 |
|  | H–4M | 700 | 700 | 700 | 400 | 700 | 700 |
| ^z^ L, M, and H in treatment codes indicate daily PPFD of 100, 400, and 700 μmol m^−2^ s^−1^ respectively.  ^y^ The basal daily PPFD during the treatment period was 100 μmol m^−2^ s^−1^, and the daily PPFD in the 6th day was 700 μmol m^−2^ s^−1^. | | | | | | | |

| **SUPP. TABLE 3 \|** Minimum root mean squared error (RMSE) between measured and estimated values from regression models of light acclimation parameters of cucumber leaves on average PPFD (*L*_m_) or time-weighted average PPFD ^z^ (*L*_w_) | | | | | | |
| --- | --- | --- | --- | --- | --- | --- |
| Parameters | Minimum RMSE | | | | | |
|  | *L*_m_ | *L*_wl_ | *L*_wq_ | *L*_wsd_ | *L*_we_ | *L*_ws_ |
| LMA | 0.16 | 0.14 | 0.11 | 0.13 | 0.13 | 0.16 |
| Chl *a*/*b* ratio | 0.03 | 0.03 | 0.02 | 0.03 | 0.27 | 0.28 |
| *P*_nmax_ | 0.09 | 0.10 | 0.08 | 0.09 | 0.09 | 0.09 |
| *V*_cmax_ | 0.11 | 0.13 | 0.11 | 0.11 | 0.11 | 0.11 |
| *J*_max_ | 0.11 | 0.13 | 0.11 | 0.11 | 0.11 | 0.11 |
| ^z^ Time-weighted average PPFDs of linear (*L*_wl_), quadratic (*L*_wq_), sigmoid derivative (*L*_wsd_), exponential (*L*_we_), or saturating (*L*_ws_) type were calculated with their corresponding weight functions. | | | | | | |
